# Supplementary material for: CANDI: a web server for predicting molecular targets and pathways of cannabis-based therapeutics
Source: J Cannabis Res. 2025 Feb 27;7:13. doi: 10.1186/s42238-025-00268-w (PMC11866588; doi:10.1186/s42238-025-00268-w)
Supplement: Supplementary file 1 — Supplementary Material 1 [file 42238_2025_268_MOESM1_ESM.docx]

**CANDI: A Web Server for Predicting Molecular Targets and Pathways of Cannabis-Based Therapeutics**

Srinivasan Ekambaram,^1^ Jian Wang ^1^, Nikolay V. Dokholyan ^1,2,3,4^*

^1^ Department of Pharmacology, Penn State College of Medicine, Hershey, PA17033, USA

^2^ Department of Biochemistry & Molecular Biology, Penn State College of Medicine, Hershey, PA 17033, USA

^3^ Department of Chemistry, Penn State University, University Park, PA 16802, USA

^4^ Department of Biomedical Engineering, Penn State University, University Park, PA 16802, USA

* Correspondence: dokh@psu.edu

**Supplementary information**


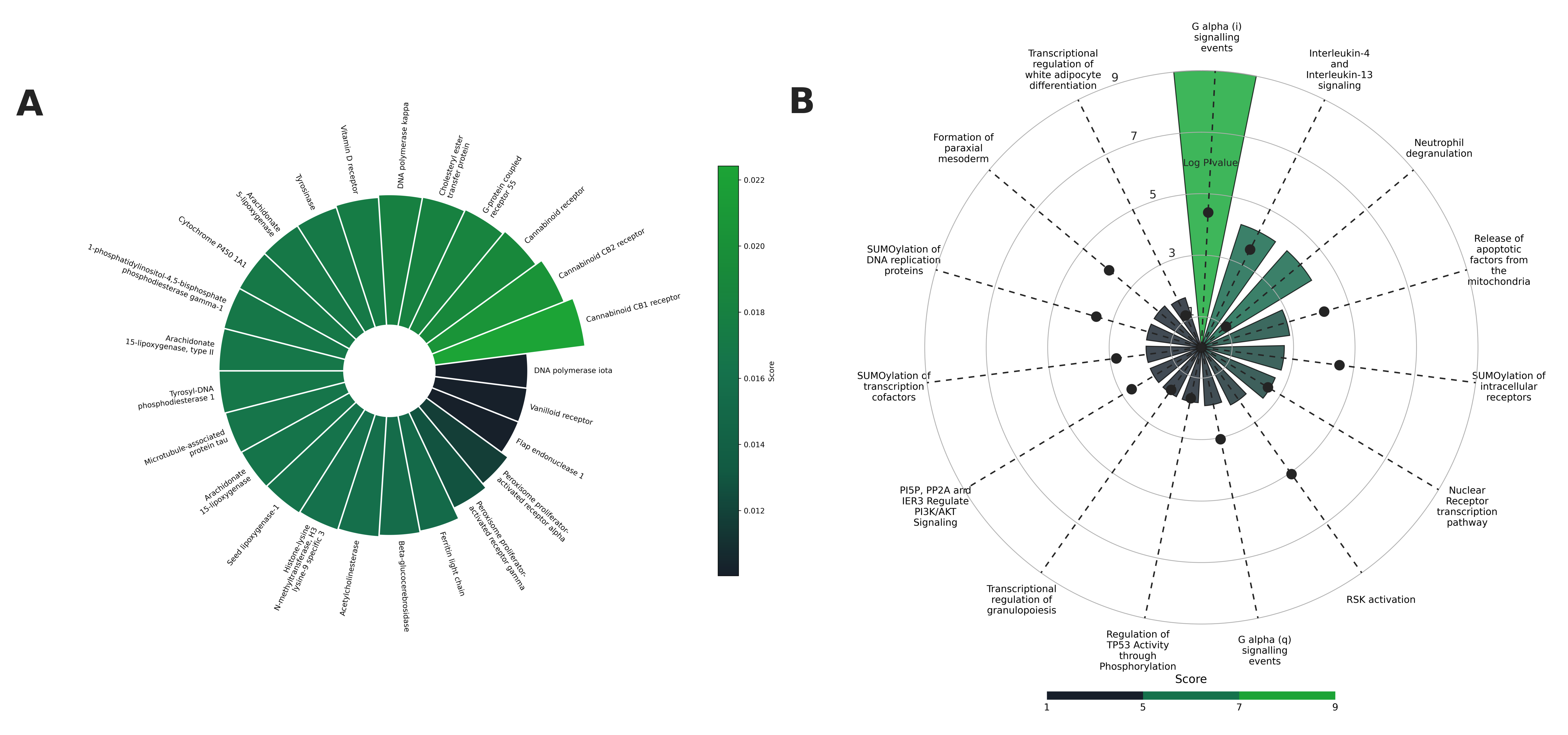


Fig.1 A case study on cannabis oil with only cannabis compounds. (A) The predicted targets for the cannabis oil formulation were ranked according to the predictive scores suggesting that CB1 and CB2 are top targets for the given formulation (B) Mapped pathways for the targets predicted elucidating the involvement of the targets in G alpha (i) signalling events regulation pathways.


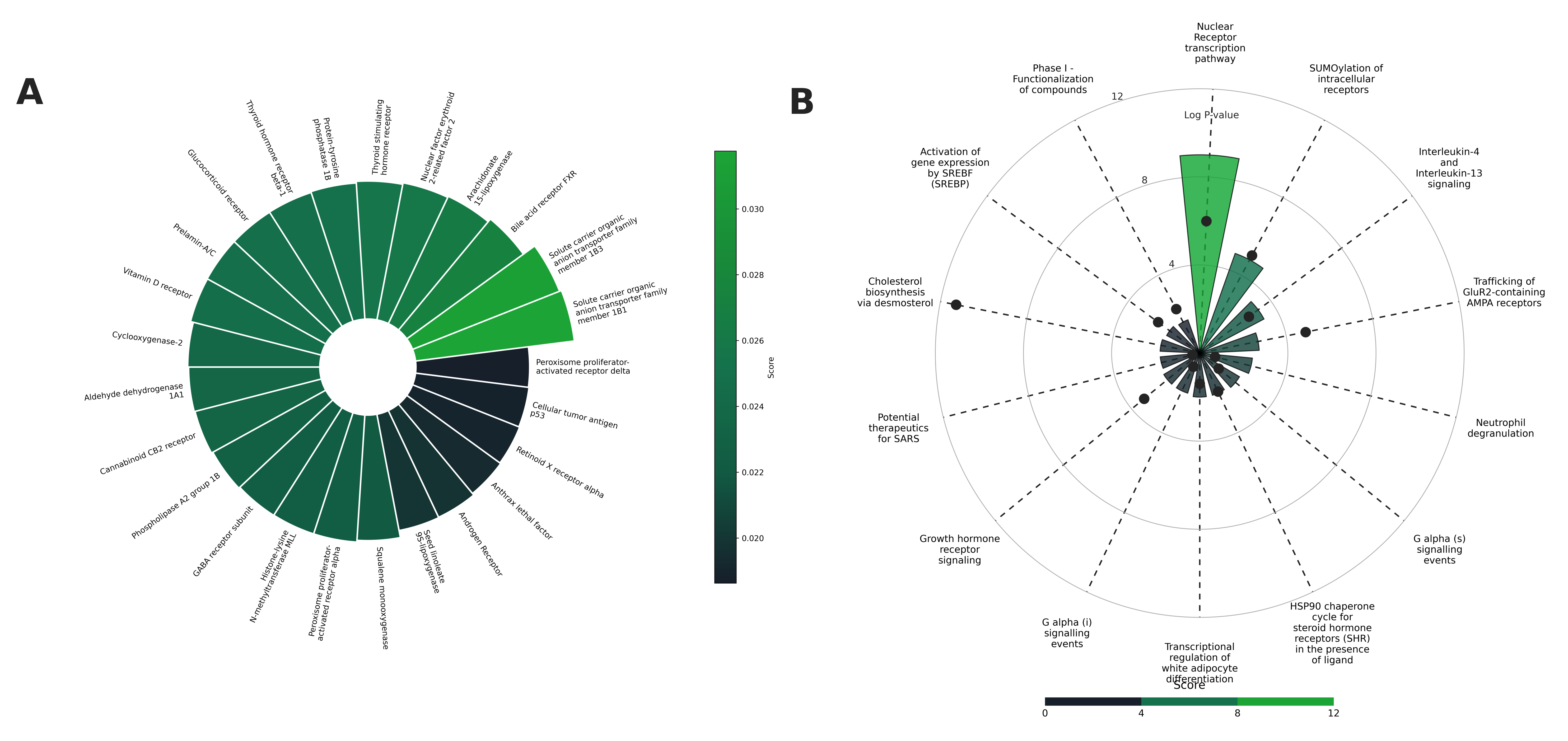


Fig.2 A case study on cannabis oil with terpenes compounds only. (A) The predicted targets for the cannabis oil formulation were ranked according to the predictive scores for the top targets for the given formulation (B) Graphical representation of the mapped pathways for the targets predicted.
